# Pathogenesis

**Published:** 1869-02-15

**Authors:** 


### *Pathogenesis.*

### CONTAGION OF PHTHISIS PULMONALIS.

La *Gazette Médicale* of Paris publishes the following paper read by M. Chauveau before the Academy of Medicine, entitled: *Application of the knowledge of the conditions of infection to the study of the contagion of phthisis pulmonalis.*

*Demonstration of the virulence of tuberculosis by the effects of the ingestion of tuberculous matter into the digestive canal. Corollaries relating to public and private hygiene.*

Impressed with the idea that if tuberculosis is a virulent affection, it should be capable of communication, almost infallibly, by the digestive canal, by reason of the exceptional facilities which would exist for the administration in enormous quantities, of the vehicles of the virus.

M. Chauveau has undertaken some experiments designed to determine this important point, and primarily he desired that the experiments should be upon a species of animals in which tuberculosis was a very common natural disease, characterized by all the gravity which it presents in the human species. His choice was therefore fixed upon the bovine species. In order not to experiment upon subjects already infected, he selected young animals; three heifers of from six to twelve months old, born and reared in freedom from all the conditions favorable to the natural development of phthisis, and a fourth heifer likewise healthy and designed to serve as a standard of comparison, not being intended to be submitted to experiment.

By the aid of a bottle and by little mouthfuls, about thirty grammes of tuberculous matter, diluted with water, and extracted from the lungs of an old phthisical cow, at the termination of variable periods, but not exceeding three weeks, these animals fell into a dying condition, after having presented all the evident signs of tuberculous affection. They were sacrificed fifty-two days after the beginning of the experiments, and at the autopsies beautifully developed lesions of generalized tuberculosis were found, with extremely marked predominance in the mesentery and the intestine.

The lungs were studded with tuberculous masses in a crude state, whose volume varied between that of a pea and that of a large filbert. As to the heifer used as a standard of comparison, although living in the same hygienic conditions she remained unaffected. The principal conclusion which M. Chauveau draws from these facts, is that "animals of the bovine species contract tuburculosis by digestive infection, as they take charbon and vaccine, as the sheep takes foot-rot, as the solipeds take glanders, as man takes variola, etc., etc."

The author concludes by indicating the important consequences which are deducible from these experiments:

*1st.* They place beyond all doubt the virulence and the contagious properties of tuberculosis, and demonstrate that the labors of M. Villemin upon the subject have not been recompensed as they deserve.

*2d.* The digestive tube in man constitutes, as in the bovine species, an avenue for contagion most appropriately arranged for the propagation of tuberculosis, and which may be much more frequently in operation than the pulmonary tract.

*3d.* If bovine tuberculosis belongs to the same species as human tuberculosis, there exists in alimentation by butcher's meat taken from phthisical animals a permanent danger to public health, a danger to which the army and the poor classes are exposed, and against which it would be important to establish measures of sanitary police.

M. Colin :—I desire to speak one word in reply to the communication of M. Chauveau relative to the transmission of charbon and of tuberculosis by the digestive canal. Three years ago I made experiments upon dogs, giving them to eat the flesh of sheep affected with charbon, and not one of them was sick. Rabbits submitted to the same alimentation have not had diarrhœa. These experiments confirm those of Renault; so that I do not believe that charbon can be transmitted by the digestive tract. It can happen only when there may be in the mouth, or in some other portion of the digestive mucus membrane, a wound, a solution of continuity which absorbs the virus; in my opinion it is a true inoculation which is produced.

The mode of transmission by the alimentary canal would be still more inexplicable for tuberculosis. I have also made experiments upon dogs and upon rabbits. I fed to a dog an entire lung which contained not less than two or three kilogrammes of tuberculous matter; he suffered nothing therefrom, so that I see in M. Chauveau's fact only a simple coincidence. Animals sold for experiment have frequently a form of phthisis, intestinal phthisis. The diges-mucous surface, and the patches of Peyer are stuffed with tubercles; a smaller number are found in the lungs. On the other hand the animal observed by M. Chauveau became phthisical only twenty days subsequent to the ingestion of the tuberculous matter. Now this is not characteristic of the progress of tuberculosis; this is not what happens, for example, when tubercle is inoculated into the cellular tissue. Indeed, several weeks are necessary to see the phthisis develop itself. Therefore, I do not believe that tuberculosis is transmissible by the digestive canal.

M. Chauveau:—I am astonished at the objections of M. Colin; they show that he has not heard the reading of my paper, and that he is not acquainted with the experiments of Renault. My paper, in fact, comprised three facts, and not one alone; the animals submitted to experiment were young heifers and not old cows; they were healthy and not sick. The coincidence is doubtless possible, but it would not be probable. I shall leave the Academy to decide this point. Relative to the transmission of charbon by the digestive tract Renault at first made negative experiments, it is true, upon dogs: but it is well known that this animal is refractory to diseases of this character (charbon). In 1851, in a work communicated to the Academy of Science, Renault stated that he had undertaken similar experiments upon herbivora, the horse, the sheep and the ox, and he affirms that he had transmitted charbon to these animals, likewise. Other experiments had conducted him to the same results with the virus of glanders.

